# Going to the South

**Published:** 1854-04

**Authors:** 


### GOING TO THE SOUTH.

HAVING been alone in the following opinions, I feel not a little gratified in inserting here a letter, written some time ago, for that ably conducted paper, the New York Observer, by one who seems to have formed his opinions from what he saw, and to have taken a common sense view of the subject, not being himself a physician. It often happens, that the most important discoveries are made by persons not naturally in the line of them.

"New Orleans, March 21, 1851.

"The climate of New Orleans, owing to the position of the city, and particularly during the winter months, is damp and exceedingly variable, the same weather seldom remaining unchanged in winter for more than three days. Since the 12th of December, the thermometer has not fallen below the freezing point, but the range above that has often been very great within a few hours. Indeed, I have never known more sudden or greater changes in any climate, than I have experienced here. I speak of the climate simply to discharge a duty, in saying that this is not the place for invalids to resort to in quest of health during the winter months, and particularly for those who are suffering from pulmonary disease. Some classes of invalids may be benefited by a residence here, and those whose lungs are but slightly affected, are frequently relieved, or entirely restored, by spending a winter in New Orleans; but where disease of this nature has become serious, and particularly in its more advanced stages, the climate of this region has a decided tendency to precipitate a fatal termination. I have known many, who came here with the hope of having a radical cure effected, whose disease has been aggravated by the change, and in some cases death has hurried them to the tomb precipitately. The climate is not only damp, but relaxing to the system, and there is such a tendency to diarrhœa, particularly in the use of the river water, that consumptive persons, having this latter tendency already fastened upon them by disease, are liable to run immediately down. In this opinion of the influence of this climate upon those who are suffering from pulmonary complaints, particularly from the consumption, I am confirmed by the views of many of the ablest dhysicians resident here, and I feel, that I am but performing an act of humanity in expressing it.

"In my sojourn here, I have met with so many sad cases of those who are sick and suffering, far away not only from the endearments, but also from the comforts of home, that I am more and more confirmed in the opinion that I have long entertained, that it is far better, as a general thing, for advanced invalids to remain at home, than to wander away, and be sick, and perhaps to die among strangers. Many are the couches by which I have stood this winter, in the discharge of ministerial duty, when the patients have sighed with bitter tears for a mother's heart, and a sister's hand to be near them, and where the only request of an earthly nature they have desired me to make in prayer for them, has been, that they might live to reach home. I have always admired, from my heart, the beauty of the Eastern salutation, '*May you die among your kindred,*' but I have never known so much of its beauty as now. It is true, heaven is as near to one place as another, and if we are prepared to enter it through the grace of our Redeemer, when once the last scene is o'er, it matters little to one who is gone, where, or in what circumstances, the last agony was endured; but there is much suffering before this hour arrives, and it leaves a lasting and bitter regret in the hearts of surviving friends, that they were able to do nothing to cheer the last hours of those who have been tenderly beloved. Unless there is a very strong ground to hope for actual restoration by a change of climate, I would advise any actually suffering invalid to remain at home. It has comforts, and palliatives, and anodynes, which are not to be found among strangers, in the most genial clime on earth. EUSEBIUS."

